# Advanced Evanescent-Wave Optical Biosensors for the Detection of Nucleic Acids: An Analytic Perspective

**DOI:** 10.3389/fchem.2019.00724

**Published:** 2019-10-25

**Authors:** Cesar S. Huertas, Olalla Calvo-Lozano, Arnan Mitchell, Laura M. Lechuga

**Affiliations:** ^1^Integrated Photonics and Applications Centre, School of Engineering, Royal Melbourne Institute of Technology University, Melbourne, VIC, Australia; ^2^Nanobiosensors and Bioanalytical Applications Group, Catalan Institute of Nanoscience and Nanotechnology (ICN2), CSIC and the Barcelona Institute of Science and Technology, CIBER-BBN, Barcelona, Spain

**Keywords:** biosensors, plasmonics, silicon photonics, microfluidics, nucleic acid analysis, epigenetics, surface chemistry, clinical diagnosis

## Abstract

Evanescent-wave optical biosensors have become an attractive alternative for the screening of nucleic acids in the clinical context. They possess highly sensitive transducers able to perform detection of a wide range of nucleic acid-based biomarkers without the need of any label or marker. These optical biosensor platforms are very versatile, allowing the incorporation of an almost limitless range of biorecognition probes precisely and robustly adhered to the sensor surface by covalent surface chemistry approaches. In addition, their application can be further enhanced by their combination with different processes, thanks to their integration with complex and automated microfluidic systems, facilitating the development of multiplexed and user-friendly platforms. The objective of this work is to provide a comprehensive synopsis of cutting-edge analytical strategies based on these label-free optical biosensors able to deal with the drawbacks related to DNA and RNA detection, from single point mutations assays and epigenetic alterations, to bacterial infections. Several plasmonic and silicon photonic-based biosensors are described together with their most recent applications in this area. We also identify and analyse the main challenges faced when attempting to harness this technology and how several innovative approaches introduced in the last years manage those issues, including the use of new biorecognition probes, surface functionalization approaches, signal amplification and enhancement strategies, as well as, sophisticated microfluidic solutions.

## Introduction

Nucleic acids (NA) have a key function in many important cellular mechanisms, such as cell differentiation, cell-division cycle, signal transduction, or metabolism (Fatica and Bozzoni, 2014; Mens and Ghanbari, 2018; Mori, 2018). Our DNA is the best-known NA molecule and it contains our genetic information. It is translated into proteins by the expression of messenger RNA (mRNAs). However, this translation may be more complex than faithful transcription of the DNA code to form proteins. Indeed, many species have cellular mechanisms that can edit the mRNAs by alternative splicing processes, resulting in a significant increase in protein diversity (Liu et al., 2012). The gene regulation process is made even more complex by the existence of a series of epigenetic mechanisms which produce a phenotypic alteration in the expression of our genome while keeping intact the DNA code. Among these epigenetic processes can be found: the methylation of the DNA, nucleosome repositioning, post-translational modification of histones, and post-transcriptional gene regulation by non-coding RNAs (nc-RNAs), such as micro-RNAs (miRNAs) (Yang et al., 2018). This set of genetic and epigenetic mechanisms results in a highly-structured gene regulatory network that serves the cells to meet their specific function and create opportunities to overcome environmental changes within the lifetime of an individual organism.

In recent years, the analysis of NA for diagnostic purposes have received special attention because of their prospective application as biomarkers for the early and accurate detection of infectious diseases (Fournier et al., 2013) and the molecular diagnosis of a great number of clinical disorders, and in particular cancer (Gingeras et al., 2005; Asadollahi et al., 2010; Lo et al., 2010; Jansson and Lund, 2012; Benesova et al., 2013; Oltean and Bates, 2013). Fortunately, NA biomarkers have been found to circulate in diverse biological media, including serum, plasma, saliva, and urine (Pös et al., 2018), so they can be targeted in the development of new diagnostic approaches. For example, circulating NAs are employed as target biomarkers in liquid biopsies in substitution of the hazardous tissue biopsies. This could overcome current clinical limitations in encompassing the cancer cell genome and epigenome heterogeneity over time since a liquid biopsy allows the recurrent monitoring of blood samples in a less invasive manner. The information gathered from these biomarkers can provide better insight into the evolutionary dynamics of cancer. In fact, circulating tumor DNA (ctDNA) seems to be a better indicator of the patient tumor status when compared with clinical serological markers (Sanmamed et al., 2015; Chang et al., 2016; Girotti et al., 2016). Other circulating NAs such as mRNA and miRNA offer other level of information (Climente-González et al., 2017; Anastasiadou et al., 2018; Koch et al., 2018; Gai and Sun, 2019). Recent studies indicate that there exist collaborative activities between these gene regulating pathways that results in a common outcome (Murr, 2010; Garcia-Gomez et al., 2018; Hanly et al., 2018). On the other hand, NA molecules from bacteria and viruses are commonly employed as markers indicative of the presence of pathogenic microorganisms in infectious disease diagnosis (Fournier et al., 2013). However, rather than simply attempting to identify foreign NA, more insight may be gained by investigating the different genetic/epigenetic mechanisms which can be affected by the infection, potentially improving diagnosis by more precisely determining infection status and progression or drug susceptibility (Bierne et al., 2012; Ciarlo et al., 2013; Kathirvel and Mahadevan, 2016). In addition to clinical diagnostics, understanding the dynamics of these networks could positively help and transform current medicine by the development of new therapies where it may be possible to revert the altered cellular process back to its normal state. For example, novel treatments based on targeted therapy are being developed for melanoma patients that harbor a BRAF mutation (Spagnolo et al., 2015). Also, RG108, which is an inhibitor of DNA methylation enzymes, can suppress radio-resistance in esophageal cancer patients (Ou et al., 2018). Likewise, several small molecules that regulate RNA splicing have been acknowledged as good drug candidates for treatment (Salton and Misteli, 2016), and personalized RNA-based therapy has been proposed to decrease tumor growth in pancreatic cancer (Gilles et al., 2018).

The most widely applied NA detection methods are those based on Polymerase Chain Reaction (PCR) or sequencing techniques (Cheng et al., 2018; Elazezy and Joosse, 2018; Zhao, 2019). PCR-based approaches allow the examination of small quantities of material with high sensitivity in a time range from few minutes to hours, which has greatly promoted the clinical analysis of NA molecules. PCR-based approaches have enabled the identification of specific NA sequence variations, such as: point mutations or single nucleotide polymorphisms (SNPs) (Zonta et al., 2016); DNA methylation (Fackler and Sukumar, 2018); alternative splicing variants (Harvey and Cheng, 2016); miRNAs (Wang et al., 2019); as well as bacterial resistance (Schmidt et al., 2019). On the other hand, next generation sequencing (NGS) methods can perform genome-wide or exon-wide analysis by massive parallel sequencing, allowing the screening of copy number aberrations (Belic et al., 2016) or SNPs (Gale et al., 2018). They have also been employed for the identification of methylation sites (Wen et al., 2015). Sequencing approaches enable to detect sequence variations that may take place during the cancer treatment cycle without any previous evidence of the primary tumour's genome. In general, the application of these methodologies for a rapid and efficient population screening still face some limitations, such as the time to complete the analysis, which usually ranges from days to hours for DNA sequencing and PCR-based, respectively, and the large volumes of sample required (Bellassai and Spoto, 2016).

Considering these facts, efforts should be directed into the development of new technologies that enable the efficient detection of genetic and epigenetic biomarkers involving simpler protocols and lower reagent and sample consumption, which will greatly reduce the cost and the time-to-result. In this context, label-free optical biosensing devices offer a full range of attractive alternatives due to their high-sensitivities and label-free detection approaches, which can be further exploited thanks to their versatility and capabilities for multiplex detection. In particular, evanescent-wave biosensors have achieved great progress for NA analyses (Carrascosa et al., 2016). They have been benefited from improvements in biosensor fabrication and production quality (Fernández Gavela et al., 2016; Soler et al., 2019), the availability of new surface chemistry methods (Escorihuela et al., 2015; Escorihuela and Zuilhof, 2017; Bañuls et al., 2019), the availability of highly efficient probes for NA detection (Shi et al., 2015; Nafa et al., 2016; Aviñó et al., 2019a), and new approaches for the enhancement of the detected signal (Guo et al., 2015). Also, the biosensor integration with microfluidics permits the incorporation of different modules, including fluidic transportation, sorting, mixing or separation methods for liquid samples, and the automation of the complete analysis, which pave the way for the full development of the so-called lab-on-a-chip (LoC) platforms (Jung et al., 2015; Szydzik et al., 2015, 2017).

The objective of this work is to give a comprehensive overview of the analytical strategies that employ evanescent-wave optical biosensors to deal with the complexities and challenges of effective NA detection, with applications ranging from identification of SPNs and epigenetic alterations in cancer, to the detection of indirect modifications of NA processes caused by bacterial infections. We also identify the main challenges faced by this optical biosensor technology and describe several innovative approaches in order to manage the existing challenges limiting the performance of the current methods.

## Evanescent-wave Optical Biosensors

**Figure 1:**
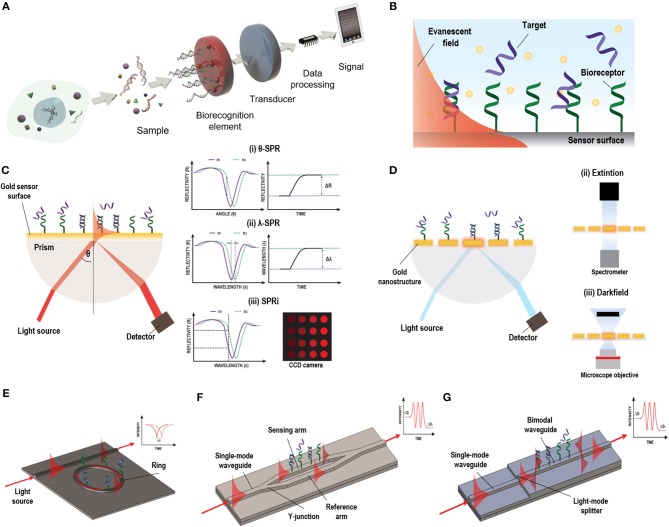
Different label-free optical biosensor based on evanescent wave. **(A)** Scheme of a biosensor. Reprinted from Carrascosa et al. (2016), Copyright (2018), with permission from Elsevier. **(B)** Evanescent field principle. **(C)** SPR biosensor based on Krestchman configuration and different detection modes: (i) fixed-angle, (ii) fixed wavelength, and (iii) fixed angle and wavelength (SPRi). **(D)** LSPR light coupling methods: (i) prism, (ii) extinction, and (iii) dark field. **(E)** Micro-ring resonator, **(F)** MZI biosensor, and **(G)** BiMW biosensor designs and working principles.

A biosensor is a self-contained device capable of providing specific quantitative or semi-quantitative analytical information using a selective biological or biomimetic recognition element (biological receptor) which is in direct spatial contact with a physical transducer (Soler et al., 2019) (Figure 1A). Optical transducers are one of the most commonly used for NA detection (Bora, 2013). They detect biological interactions by evaluating the variations induced in the light properties, such as intensity, wavelength, refractive index, or polarization (Sang et al., 2016). They can be classified in label-based or label-free sensors. Label-based usually detect changes in color or the presence of photons generated at a particular wavelength by optical labels, such as dyes/DNA intercalators or fluorescent molecules. The label serves as an indirect indicator of the presence of a concrete analyte. Hybridization between a DNA probe and its specific target has been detected by doping with chromophore molecules (Szukalski et al., 2019). Fluorescence emission from molecular beacons (Yao et al., 2003), quantum dots (Chen et al., 2010a), or fluorescent-labeled signal probes (Zhu et al., 2017) have also been employed for NA detection. Although the use of optical labels is widespread in NA analyses, these methods are inherently time-consuming and can greatly increase the costs of the assays (Chen et al., 2017). In addition, they are prone to sample losses in the labeling process and, in the case of fluorescence, fluorophores are quite sensitive to environment conditions, such as pH, and can be bleached very fast, reducing the efficiency of the analysis (Sang et al., 2016).

On the other hand, label-free optical biosensors enable measurement of the physical properties of the analytes under a label-free scheme, removing experimental ambiguity, and allowing for more reliable analysis that require minimal assay development (Chen et al., 2017). There is a vast repertoire of label-free optical biosensors able to transform a specific biological interaction into a quantifiable signal based on different working principles, including the scattering of light or the generation of an evanescent wave. Evanescent-wave biosensors have been recognized as ideal candidates for NA evaluation without the need of any molecular marker (Carrascosa et al., 2016). They exploit the ability to confine an electromagnetic field within or at the surface of dielectric and/or metal structures in the form of localized or propagating electromagnetic modes with well-defined optical properties. Part of the electromagnetic mode extends beyond the surface into the external medium, as a so-called evanescent field (Figure 1B). Variations in the refractive index of the external medium just above the surface will cause a change of the optical properties of the excited optical mode through this evanescent field, resulting in a variation of its effective refractive index and allowing for a quantitative measurement. Due to the exponential decay of the evanescent field in the external medium (usually few tens to few hundred nanometres), only changes occurring in close proximity to the surface will be sensed, with a natural filtering of background from the surrounding medium. Evanescent detection does not become weaker with reduced sample volume, instead it is proportional to the concentration of analyte that come into contact with the functionalised surface. This feature makes evanescent wave biosensors particularly attractive for use with reduced sample volumes and consequently reduced auxiliary reagent volumes as well.

Evanescent-wave biosensors enable monitoring of biomolecular interactions in real-time opening the possibility of evaluating the affinity and kinetics of the interaction and can contribute to a more concrete disease diagnosis. These type of biosensors also benefit from versatility—theoretically, it would be possible to selectively sense an almost limitless range of analytes just by selecting the appropriate biological receptor. Currently, new options for sensor transducers are emerging due to the progress in nanofabrication technology which further provide interesting opportunities for miniaturization, high-throughput and low-cost production (Sagadevan and Periasamy, 2014; Sang et al., 2016). Plasmonic and silicon photonics based biosensors are among the most employed evanescent-wave biosensors for the analyses of NAs with potential applicability in clinical diagnosis (Ermini et al., 2014; González-Guerrero et al., 2016). These biosensors have shown high detection sensitivities with short response times. In this review, we will identify the main challenges faced with plasmonic and silicon photonic biosensors and summarize some of the most recent analytical strategies developed for the analysis of NA biomarkers with these devices.

### Plasmonic-Based Biosensors

Plasmonic-based biosensors constitute the most employed evanescent-wave optical biosensors for NA analyses. Among them, Surface Plasmon Resonance (SPR) biosensor is the most advanced and mature technology (Soler et al., 2019). SPR biosensors employ propagating surface plasmons that oscillate collectively on a planar metal-dielectric interface. The surface plasmon electromagnetic field propagation is constrained vertically at this interface but is free to propagate laterally along the film. The plasmon wave propagates with very specific phase and attenuation properties which depend strongly on the dielectric environment. The plasmon mode has a strong evanescent field just above the surface of the metal facilitating biosensing. For the plasmon excitation, a light source should be effectively coupled to a thin metal layer, which is usually a 45–50 nm thick film of gold. There are several configurations for light coupling in SPR sensors to promote the excitation of the surface plasmons, including prism coupling, waveguide coupling, and grating coupling (Soler et al., 2019). Among these, the prism-based Krestschmann configuration harnessing Attenuated Total Reflection (ATR) is the most widely employed (Figure 1C). In this type of configuration, the surface plasmon is manifested by a drop in the intensity of the light reflected, that strongly depends on the refractive index of the dielectric. This surface plasmon can be tracked to study any molecular interaction taking place at the gold sensor surface by looking at changes in the coupling angle of incidence or in the coupling wavelength. Moreover, in SPR imaging (SPRi) systems, a multiplexed array format can be achieved by fixing both, the wavelength or the angle of the incident light, and measuring the intensity of different spots using a CCD camera (Wong and Olivo, 2014). SPR biosensors offer high sensitivities ranged between 10^−5^ and 10^−6^ refractive index units (RIU), corresponding to NA detection limit within the pM-nM range with optimal biofunctionalization conditions. Currently, there are already many different SPR systems commercially available which are broadly deployed in industry, hospitals, or academia (Prabowo et al., 2018). However, they still involve bulk components and specialized procedures, increasing their prices and complexity rendering them not affordable for clinical setting. The new trends aim at reducing the components' costs, including novel light source technology and detectors by harnessing current technology that can be easily found everywhere, such as smartphones, to decrease the costs and foster the portability of such biosensor devices (Liu et al., 2015c).

Parallelly, in the last decade, with the progress of nanotechnology, a new generation of plasmonic sensors has been introduced that can improve miniaturization, multiplexing capabilities, and biosensor chip integration. These biosensors use more sophisticated nanostructured metal films or so-called nanoplasmonic structures with features on the scale of the wavelength of the incident light or even smaller. In these nanoplasmonic structures, the surface plasmon can be confined in all three dimensions inducing the non-propagating collective oscillation of the metal free electrons termed localized surface plasmon resonance (LSPR). This confinement provokes an increment in the electromagnetic field near the structures that rapidly falls off with distance and minimize LSPR biosensors susceptibility to variations produced in the external media by, for example, changes in temperature, and enhance their sensitivity toward small analytes found at very small concentrations. Sensitivity can be enhanced by an appropriate design of the nanostructures, which can be exploited to promote different resonance modes. In fact, these resonances can be modulated to show highly reproducible strong nearfield enhancement and sub-wavelength light confinement that enable to detect specific molecular fingerprints (Dong et al., 2017). The simplicity of the coupling of the light has fostered different detection approaches (Figure 1D). Also, the facile device miniaturization provides better opportunities for multiplexed device formats, highlighting the superiority of nanoplasmonic biosensors over conventional SPR ones. Furthermore, the performance of the nanostructures during the detection of NAs can be predicted by the employment of an universal model by taking into account the optical and mass transport aspects, encouraging the development of devices with maximized capabilities (Špačková et al., 2018).

### Silicon Photonic Based Biosensors

Silicon Photonics biosensors are gaining momentum in the diagnostic field. They consist of waveguides (i.e., structures of conductive or dielectric material used to guide high-frequency waves, such as electromagnetic waves in the case of optical waveguides) that confine light in vertical and horizontal dimensions but enable the propagation of light in a longitudinal dimension with minimal loss of energy. Although optical waveguides are normally used in integrated photonic circuits as an optical analogy to electrical wires, they can be also employed as high sensitive transducers in biosensor devices. These waveguides are highly compact and can be easily miniaturized and patterned in complex forms with extremely predictable behaviors. This offers the opportunity to generate arrays of sensors within the same photonic chip for multiplexed analysis. Silicon photonics sensors are fabricated using conventional silicon microfabrication techniques, including photolithography and etching processes. This kind of fabrication allows for wafer-level packaging, producing numerous sensors in the same fabrication process with accurate precision and reproducibility, reducing the time and the manufacturing costs (Fernández Gavela et al., 2016). They have good optical properties, high stability with temperature, inertness to many chemicals and solvents, low surface roughness and a versatile chemical functionalization (Escorihuela et al., 2014b). In silicon photonic biosensors, the light is confined within a waveguide. The propagating light modes (guided light modes) also exhibit an evanescent field outside the waveguide surface. This portion of the evanescent field of the guided light modes is sensitive to refractive index changes at the sensor surface, which will produce a variation of the phase velocity of the propagating mode. Several architectures have been implemented for biosensing, among which optical resonators and interferometers have been extensively employed for the analysis of NA biomarkers (Carrascosa et al., 2016).

The most common optical resonator in the recent literature is the micro-ring resonator. The main advantage of micro-ring resonator-based biosensors reside in their compactness, the ease of design and robust fabrication (Ciminelli et al., 2019). In a conventional ring resonator arrangement, the light passes through a straight waveguide which is coupled to a closed-loop waveguide (micro-rings) (Fernández Gavela et al., 2016) (Figure 1E). The surface of the ring structure is uncovered, generating an evanescent field. In a ring resonator, the sensitivity will increase depending on the number of round-trips supported by the micro-ring, which is defined by the quality factor Q. The biosensor intensity builds-up by multiple interactions with the external environment and has reported sensitivities up to 10^−6^–10^−7^ RIU.

In interferometers, light is generally divided in two light waves, one employed as a reference and the other one for sensing. Only the sensing-light wave will be susceptible to variations of the refractive index, which can be detected at the sensor output. Technologies based on light interferometry appear to be the most sensitive platforms reported. One of the most common configuration employed for biosensing is the Mach-Zehnder Interferometer (MZI) (Zinoviev et al., 2008). In its basic configuration, MZI has an input single-mode waveguide with power equally split between two arms by a Y-junction. One of the two arms is exposed to the external medium to be sensed, while the other is employed as a reference (Figure 1F). The two arms are recombined through a second Y-junction into a single output waveguide, resulting in an interference pattern with a determined number of fringes of a certain amplitude (visibility factor) depending on their sensitivity, which has been demonstrated to be 10^−7^ RIU. However, the design and fabrication of the MZI should be optimized to achieve a symmetric splitting and recombination of the light, as well as balanced losses on both the sensing and reference arms. The BiModal Waveguide (BiMW) interferometer has been proposed as an alternative to this configuration since it avoids light beam splitting and recombination (Zinoviev et al., 2011). In this interferometer, rather than the sensing and reference beams propagating along physically separated arms of the MZI, sensing is achieved by comparing two different supported modes (fundamental and first order light modes) within the same straight waveguide (Figure 1G). This results into a device with improved tolerance to fabrication variations and a smaller footprint, opening the possibility to fabricate more sensors in the same area, with a consequent increase in the reproducibility and reliability of the sensing evaluations. In the BiMW, the first order mode is far more evanescent than the fundamental mode. Hence, during the biointeraction event, the fundamental mode would act as the reference wave being only weakly influenced by the biointeraction, while the first order mode will be strongly influenced by the refractive index changes occurring within the evanescent field. The interference of both modes produces a phase variation signal similar to the MZI, which is collected at the end of the waveguide. This configuration has demonstrated a bulk sensitivity of 10^−8^ RIU, becoming one of the most sensitive interferometers described in the literature for label-free direct detection of NAs (Huertas et al., 2016b, 2017). Silicon photonic biosensors based on MZI and BiMW are totally integrated devices that include the light beam splitting and recombination, providing further stabilization to mechanical vibrations (González-Guerrero et al., 2016). However, the interferometric signal has a sinusoidal trend and can lead to ambiguity during signal interpretation. These drawbacks been overcome at the expense of post-processing of the read-out signal with the use of different innovative forms of modulation based on all-optical phase modulation (Dante et al., 2012) or optical frequency combs (Knoerzer et al., 2019), improving the performance of these integrated photonic biosensors.

Further advances in the complete integration of the optical components into the same silicon chip is being pursued in order to satisfy the necessity of point-of-care systems for their use in clinical settings or for healthcare diagnosis applications. Auxiliary equipment such as signal acquisition devices or high current power supplies occupy significant volume of space, which detracts from the benefits provided by the micro/nano scale of these devices. Efforts are directed to the integration of multiple functions, such as on-chip lasers. Different materials, either alone or in hybrid approaches, have been employed for the integration of lasers in silicon chips, such as germanium (Wirths et al., 2015; Margetis et al., 2018), or group III/V materials (Roelkens et al., 2015). While the former increases the power supply of the biosensors, the latter maximize the density of integration and, therefore, the portability (Luan et al., 2018). Because they are compatible with complementary metal oxide semiconductor (CMOS) fabrication, different detectors based on these type of materials have been also proposed (Luan et al., 2018).

### Challenges in Label-Free Optical Biosensing of Nucleic Acids

Sensitivity is one of the main challenges in the detection of NA. Not only are these biomarkers present in low concentrations in real samples, but in most cases, they are related to subtle changes in gene expression. In addition, the dynamic range of recognition usually should cover a wide range of NA concentrations, such as the case of miRNA analysis, where the over-expression or sub-expression usually cover from nanomolar (nM) concentrations to attomolar (aM) (D'Agata and Spoto, 2019). The outstanding sensitivity required for NA analysis can be obtained by the combination of highly sensitive biosensor transducers and signal pre- and/or post-amplification approaches by PCR-based methods or signal enhancers, such as nanomaterials or DNA/RNA binding proteins. However, this sensitivity not only depends on the physical transduction mechanism of these devices but is vastly dependent on the biofunctionalization approach and the quality of the selected biorecognition element (González-Guerrero et al., 2016). The bioreceptor layer must interact with sufficient specificity and selectivity with the target NA. It is highly dependent on both, the proper covalent attachment to the surface, and the hybridization event. Successful sensing must also ensure discrimination between homologous sequences that could require differentiation between a single nucleotide mismatch.

The nature of the NA is another aspect that should be considered. RNA molecules are easily degradable by RNases. Their half-life is constrained by the presence of these enzymes and the detection methodology may require the inclusion of purification steps (Baratchi et al., 2014). In addition, target lengths could also complicate the detection process. While short sequences may produce lesser sensor signal increments, thereby, worse limit of detection (LOD), long sequences are more prone to generate secondary structures by self-loop formation by base paring, and the target sequence may be hindered. In the case of DNA biomarkers, they usually are long and doubled stranded sequences, being difficult to capture. In this case, detection approaches may need to include the previous use of an enzyme restriction protocol, denaturation steps or special probe designs that enable double strand DNA (ds-DNA) capture.

The study of gene regulation pathways implies both, the detection and quantification of the NA molecules that participate in such processes, i.e., DNA fragments, mRNA isoforms generated by alternative splicing or micro-RNA regulators, and the recognition of the epigenetic markers such as the 5-methylcytosines in DNA. In addition, although independent analysis of each type of NA biomarker are proven to enhance the sensitivity and specificity of the assays in comparison other circulating biomarkers (Bratman et al., 2015), multiplexed biomarker detection will definitely help to further understand the dynamics of a concrete disease and increase the sensitivity of the diagnosis (Sullivan et al., 2019).

Finally, sample pre-treatment and manipulation are critical for NA biosensing. Direct evaluation of biofluids such as blood or urine remains challenging and is yet to be effectively solved for direct label-free evaluation (Soler et al., 2019). The high amount of proteins and lipids present in blood can adsorb onto the sensor surface and usually lead to high background signals, obscuring the specific signal of the target and hindering their recognition. Therefore, the biosensor should be provided with a low-fouling biosurface in order to prevent non-specific adsorptions. In addition, NAs are present not only in circulation, but also inside cells, exosomes, or organelles. In order to avoid cross-contamination, samples may require the separation of specific cells, RNAs, DNAs, etc. (Gai and Sun, 2019). Circulating DNA needs better plasma separation methods because current protocols include centrifugation steps that are too aggressive, promoting circulating cell disruption and, therefore, the release of undesired NA material. In addition, this extraction should be performed right after sample extraction to prevent from any contamination of DNA from circulating blood cells. Here, the integration of the optical biosensor with microfluidic lab-on-a-chip (LoC) platforms could provide less aggressive, fast and more efficient solutions.

## Nucleic-Acid Based Label-Free Optical Biosensors

In label-free optical biosensors, the biorecognition layer at the surface of the transducer is of paramount importance. The final sensitivity and specificity of a biosensor are directly related to the activity of the immobilized molecules and the accessibility of the specific targets. In the development of NA biosensors there are two key steps: (i) the biorecognition element design, responsible of the system selectivity; and (ii) the surface functionalization chemistry, which should ensure: a good surface coverage; proper accessibility of the target to the immobilized biolayer; low non-specific binding; and good reproducibility and robustness (Bañuls et al., 2013).

### Biorecognition Elements in Nucleic Acid-Based Biosensors

**Figure 2:**
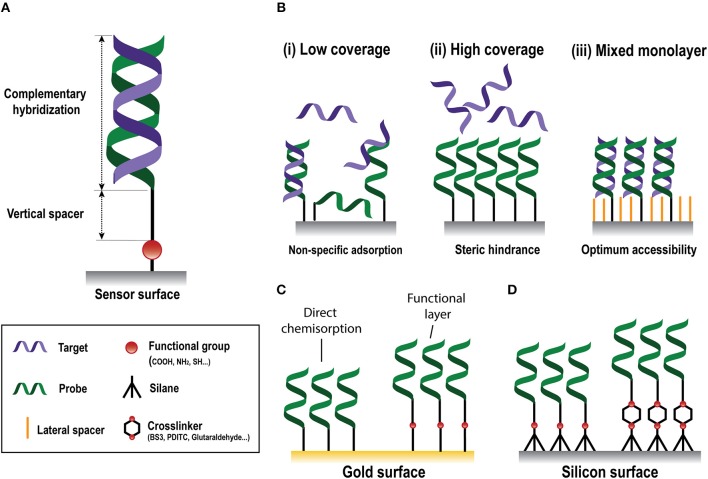
Nucleic-acid biosensors surface functionalization. **(A)** Scheme of a standard DNA probe. **(B)** Different surface coverages: (i) low, (ii) high, and (iii) mixed monolayer. **(C)** Gold surface immobilization strategies based on direct chemisorption (left) and on the generation of a functional layer (right). **(D)** Silicon surface immobilization strategies through silanes without (left) or with (right) crosslinkers.

The biorecognition element must ensure high selectivity to enable single-nucleotide resolution, homologous sequences discrimination, and negligible cross-hybridization from non-specific NA molecules present in the same sample. In NA biosensors, the conventional biorecognition element is a single-stranded DNA (DNA probe) with a specific sequence of nucleotides of around 9–50 bases, which hybridizes to a complementary NA molecule. Whereas, antibody production involves their induced expression by the injection of a specific antigen into laboratory or farm animals/cells and their eventual recovery (Chon and Zarbis-Papastoitsis, 2011), DNA is an easily synthesizable biorecognition element. Current biotechnological methods permit the *in-vitro* production of synthetic NAs with the desired sequence in large amounts and with high degree of purity (Hughes and Ellington, 2017). They can be customized depending on their application by introducing different modifications in both the 5' and the 3' ends. Thus, structural end-modifications can be introduced in the DNA probe sequence for their direct immobilization over different types of inorganic materials to generate functional surfaces for NA detection at a very low manufacturing cost. In the design of ss-DNA probes, three factors must be considered: (i) the functional group that will allow the attachment of the probe to the sensor surface; (ii) a vertical spacer to improve accessibility, and (iii) the sequence itself (Figure 2A). A wide variety of functional groups are available for synthetic oligonucleotides depending on the surface chemistry selected for the attachment. Short oligonucleotides modified by amino, thiol, hydrazide, phosphorothioates, or biotin are commonly used for DNA immobilization (Zourob, 2010). End modification of DNA probes not only introduces a site-specific group for their oriented covalent attachment, but also allows insertion of a spacer between the probes and the surface. This vertical spacer improves the mobility of the immobilized probes and their accessibility by the complementary target sequences. They also move the DNA sequence away from the sensor surface, reducing the adsorption and steric effects (Carrascosa et al., 2012). Different vertical spacers can be introduced, such as a chain of 6 or 12 carbons (C6 or C12, respectively) (Schmieder et al., 2016) or poly-thymine (polyT_m_) sequences of different lengths (Huertas et al., 2017, 2018) which acts as a vertical spacer due to the low affinity of thymine bases for gold surfaces (Opdahl et al., 2007).

For the selection of the probe sequence there are available many commercially manufactured and well-understood codes that help to tailor the probe-target stability of a given application (Ermini et al., 2011). An important challenge is the presence of regions that can assume conformations by self-hybridization and may hide the binding sequence of interest. To avoid self-hybridization, probe length and C-G content are determinant factors. Probes containing between 15 and 25 bases permit strong hybridization while avoiding self-complementarities and reducing the likelihood of cross-hybridization from undesired molecules (Ermini et al., 2011). At the same time, a 40–60% content of C-G bases promotes a stronger hybridization due to higher contribution of stacking interactions during hybridization, hence contributing to the stability of the formed hybrid (Hormeño et al., 2011). However, excessive CG content may lead to non-specific hybridization of other sequences bearing also a high quantity of these nucleotides.

In some cases, the design of the probes is restricted to a limited sequence such as the case of short NAs. This difficulty becomes even more challenging due to their high heterogeneity, since such sequences have isoform or homologous sequences with differences up to the single mismatch. In these situations, the probe design is constrained, putting at risk the sensitivity and selectivity of the biosensor. Therefore, alternative strategies should be considered. Certain buffer compositions have traditionally solved cross-hybridization problems. The stability of NA duplexes can be also compromised by the ionic strength of the solution employed for the analyses (Tan and Chen, 2006). Structural integrity of DNA has been found to be dependent on the DNA affinity for monovalent cations such as K^+^ and Na^+^ (Kielar et al., 2018). Thus, buffer cation content can be fine-tuned to obtain an appropriate selectivity. In addition, several agents can be employed in the hybridization buffer to reduce the melting temperature (i.e., the temperature corresponding to the midpoint in the transition from helix to random coil) of the hybridization, without requiring any temperature control. Formamide is a denaturant compound that has been shown to reduce the melting temperature in aqueous solutions, increasing the hybridization efficiency (Fontenete et al., 2016; Huertas et al., 2016a, 2017). Tetramethylamonium chloride (TMAC) and glycine betaine have been reported to be isostabilizing agents, altering the melting temperature and making the hybridization solely dependent on oligonucleotide length, despite their GC content (Duby et al., 2004; Napolitano et al., 2004; Schwinefus et al., 2007). The destabilizing effect of such agents increases with increasing GC content, with almost no effect on poly(dAdT) (Schwinefus et al., 2013). On the other hand, some other components, such as urea, have the opposite effect, promoting a decrease in the strength of AT/U bonds more than GC base pairs (Schwinefus et al., 2007). These agents can be employed alone or in combination in order to achieve the proper hybridization efficiency depending on the nucleotide sequence of the targets (Huertas et al., 2016b).

Enhanced target capture efficiency, and therefore, contribute to increase the sensitivity of these biosensors, can also be achieved by introducing nucleotides exhibiting restricted conformation that promote the base stacking and backbone pre-organization, such as nucleic acids (LNA) (Fontenete et al., 2016). The LNA is a ribonucleotide homolog with a characteristic 2'-O,4'-C-methylene bridge that can increase the melting temperature by 2–8°C per subunit introduced into DNA or RNA oligomer. This configuration provides higher binding affinity and foster discrimination of base mismatches, as well as minimize the risk of digestion by nucleases (Bakthavathsalam et al., 2018). Peptide nucleic acids (PNAs) are DNA/RNA analogs where the sugar-phosphate backbone is substituted by units of *N*-2-aminoethylglycine (Gupta et al., 2017). Their backbone is neutral and generates no electrostatic repulsions, allowing a remarkable stability toward complementary NAs. They are also able to interact with both, DNA (Kirillova et al., 2015) and RNA (Sato et al., 2016) duplexes and PNAs derivatives can be obtained by chemical modification for enhanced affinity (Annoni et al., 2016) and solubility (Sahu et al., 2011). Phosphorodiamidate morpholino oligos (PMO) are also synthetic DNA analogs that possess a neutral backbone of morpholine rings, which not only provides higher solubility in aqueous solutions than PNAs, but also more flexibility in length (Liao et al., 2017). Probes based on triplex-affinity capture are gaining much interest for the detection of NAs in biosensing platforms (Aviñó et al., 2019a). NA triplexes can be induced by the interaction of DNA or RNA molecules with a hairpin-like polypurine-polypyrimidine-rich sequence. They have proven to increase the hybridization efficiency of long and highly structured RNAs (Carrascosa et al., 2012) and small RNA sequences (Aviñó et al., 2016). They also show high potential for interacting with duplex DNA fragments by promoting strand displacement (Huertas et al., 2018). Functional nucleic acids (FNAs) such as aptamers have also been developed using a combinatorial method called SELEX (systematic evolution of ligands by exponential enrichment), making possible to evolve NAs in test tubes to bind to a diverse range of analytes beyond DNA or RNA with high affinity and specificity (Liu et al., 2009). Further structural modifications of these aptamers have been proven to outperform the aptamer affinity (Aviñó et al., 2019b). However, biosensor applications using this type of biorecognition elements are out of the scope of this review. Table 1 summarizes the existing types of probes used in NA biosensors.

**Table 1:** Different types of probes, main characteristics, target sequences, and advantages.

**Probe**	**Main characteristics**	**Target**	**Advantages**
DNA	Conventional sequence	Single-stranded NAs	Simple synthesis
LNA	2'-O,4'-C-methylene bridge	Single-stranded NAs	Increase melting Tª, affinity and mismatch discrimination Decrease nuclease digestion
PNA	N-2-aminoethylglycine backbone (neutral charge)	Single and Double-strand NAs	Increase hybrids complex stability
PMO	Morpholine rings backbone	Double-strand NAs	Increase solubility and length flexibility
DNA clamp	Polypurine/polypyrimidine-rich sequences Antiparallel sequences 8-aminoG modifications	Double-strand NAs and structured NAs	Increase hybridization efficiency

### Surface Functionalization for Probe Immobilization and Antifouling Coating

Physical adsorption is the simplest immobilization method. This approach does not require any NA modification taking advantage of intermolecular forces such as electrostatic, hydrophobic, and/or polar interactions (Zourob, 2010). However, this adsorption is solely governed by physical attractive forces between the biomolecule and the sensor surface, so that the amount of adsorbed DNA probes cannot be controlled and may vary along the surface, representing an important drawback for the biosensor performance. In addition, flow-through assays or changes in the pH or buffer composition can easily lead to desorption of the biomolecules (Rabe et al., 2011), causing a loss of the signal and possible cross-contamination of the surface. On the other hand, covalent immobilization approaches involve chemical reactions to link the biomolecules to the sensor surface by covalent bonds. Covalent linking generally requires the modification of the oligonucleotide probe during the synthesis process with a functional group to guarantee the appropriate chemical bonding. Chemical grafting prevents the release of probes into the solution, surpassing one of the main drawbacks of physical adsorption and promoting better sensor reproducibility. Likewise, covalent linking can reduce the background signal coming from non-specific adsorption. Different functionalization strategies for gold and silicon surfaces for the development of label-free optical NA biosensors will be introduced in the next sections.

#### DNA Immobilization Strategies for Gold Surfaces

Gold sensor surfaces are the most employed in plasmonic biosensors. Chemical adsorption (i.e., chemisorption) of thiolated molecules on gold is the most widely used immobilization approach due to its easy preparation. This approach takes advantage of the strong affinity of thiol atoms toward gold surfaces (Sakao et al., 2005). Chemisorption of DNA probes modified with a thiol linker, i.e., SH-DNA probes, is the most straightforward approach for the development of NA biosensors (Figure 2C), generating self-assembled monolayers (SAMs) carrying directly the probe sequence that will hybridize with the target. The grafting density of SH-DNA probes is high compared to those obtained using other immobilization methods (Peeters et al., 2008) and it is affected by the length of the immobilized probes (Ulman, 1996), pH (Xia et al., 2010), or the salt content of the employed buffer (Satjapipat et al., 2001). However, a highly dense and compact DNA monolayer is not always ideal, since it can hinder the accessibility of target sequences due to steric and electrostatic forces, diminishing the likelihood of hybridization and, therefore, the detection signal (Lee et al., 2006b) (Figure 2B). Hybridization signal can be enhanced by optimization of the DNA coverage and surface properties with certain small-molecules that can act as both, lateral spacers and blocking agents, such as 6-mercapto-1-hexanol (MCH) (Satjapipat et al., 2001; Lee et al., 2006b), long alkanethiols (Gong et al., 2006), or thiolated oligoethileneglycols (Lee et al., 2006a). These molecules also promote the orientation of the probe, reduce their density and improve the resistance to non-specific adsorptions (Satjapipat et al., 2001; Gong et al., 2006). The adsorption of poly-adenine (polyA) sequences to the gold surface has been proposed as an alternative to SH-DNA probes (Sohreiner et al., 2010). This approach relies on the unusually strong interaction between adenine nucleotides and gold.

The surface of an SPR sensor can also be modified with a functional layer that carries various functional end-groups, such as maleimide (Lee et al., 2007), amine (Brockman et al., 1999), or carboxyl (Burgener et al., 2000) groups for the subsequent covalent immobilization of the probes (Figure 2C). Carboxyl (-CO_2_H) groups are widely employed, and usually involves the covalent bonding of amine-modified DNA (NH_2_-DNA) probes. This covalent coupling consists of an amide bond formed between the primary -NH_2_ of the DNA probe and the -CO_2_H terminated monolayer, which is previously activated by the well-known carbodiimide-mediated chemistry. A solution containing a mix of EDC/NHS (1-Ethyl-3-(3-dimethylaminopropyl)-carbodiimide/N-hydroxysuccinimide) is employed to activate the -CO_2_H groups by producing a NHS-ester intermediate highly reactive to the primary amine in the DNA probe (Conde et al., 2014).

Other strategies make use of affinity linkers, such as biotinylated probes, which can be efficiently immobilized on avidin/streptavidin modified surfaces (Vaisocherová et al., 2006; Mir et al., 2008; Biagetti et al., 2018). The interaction between avidin/streptavidin with biotin is known to be strongest protein-ligand non-covalent bond. Once formed, it is very rapid and remains stable even under extremes pHs and temperatures, when dissolved in organic solutions, or in the presence of other denaturing agents.

#### DNA Immobilization Strategies for Silicon-Based Surfaces

In silicon-based NA biosensors, surface modification with organofunctional alkoxysilanes has received widespread application due to their considerable stability and rapid covalent linkage (Hu et al., 2015). Although silane SAM formation in silicon surfaces is not as straightforward as thiol SAM on gold surfaces, it shows higher physical and chemical stability, since it allows to implement a wide variety of chemical reactions (Bañuls et al., 2013). Silane SAMs are obtained by a chemisorption of trichloro-, trimethoxy-, or triethoxy molecules, onto the sensor surface followed by condensation of this molecules with hydroxyl groups generated at the silicon surface. The silanization reaction will be strongly influenced by the experimental conditions, where the most relevant parameters are the nature of the silane, its concentration, nature of the solvent, water content, temperature, and time (Bañuls et al., 2013). Silanes can have different functional groups which introduce the specific surface functionalization for the subsequent immobilization of the probes (Figure 2D). Amino-terminated silanes, such as APTES (3-aminopropyltriethoxysilane), is one of the most widely used for biofunctionalization due to their reactivity with different functionalities, such as aldehyde, carboxylic acid, and epoxy. APTES silane can incorporate different crosslinkers, a class of molecules capable of linking two functional groups together, e.g., surface groups and biorecognition elements, in solution. BS3 (bis(sulfosuccinimidyl)suberate), a water-soluble amine-to-amine homobifunctional crosslinker, can be employed for the covalent bonding of NH_2_-DNA probes (Cardenosa-Rubio et al., 2018). This crosslinker contains an NHS ester at each end that enable the formation of amide bonds when reacting with primary amines. Amino-silane surfaces can be also activated with 1,4-phenylenediisothiocyanate (PDITC), which provide isocyanate groups that can be reactive either to NH-DNA probes (Hu et al., 2015), yielding thiourea bonds, or to SH-DNA probes (Huertas et al., 2016b), forming thiocarbamate bonds. However, the most used homobifunctional crosslinker is glutaraldehyde, which forms a surface with aldehyde groups that lets the formation of imines by their covalent reaction with amine groups (Zainuddin et al., 2018). The fact that homobifunctional crosslinkers carry two identical chemical groups can cause undesired side effects such crosslinking between surface groups or biorecognition elements, which can actually inhibit the functionalization process. In this regard, heterobifunctional crosslinkers can overcome those side-effects due to the different nature of their reactive groups (Jin et al., 2003). Other silanes carry SH- as the functional groups, such as MPTS (Mercaptopropyltriethoxysilane). Thiolated monolayers allow rapid and simple immobilization of SH-DNA probes by different chemistries without using any crosslinker or other reagent, such as disulphide bond linkage (Sánchez del Rio et al., 2007), or through thiol-ene *click* photochemistry (Escorihuela et al., 2014a). The latter one can also be also employed by activation of the thiol group at the probe to further reaction with different functional groups at the sensor surface, such as alkenylated and alkynylated surfaces by forming thiol-ene and thiol-yne links, respectively (Bañuls et al., 2019). These approaches can be enhanced by the use of polythiolated probes, generating multiple anchor points at the sensor surface (Bañuls et al., 2017).

#### Generation of Antifouling Monolayers

The performance of label-free optical biosensors may be compromised by interfering effects that produce refractive index changes unrelated to the analyte binding, referred to as fouling effects (Vaisocherová-Lísalová et al., 2016). When the target molecules are contained in a complex solution, the specific response due to their capture may be concealed due to the adsorption of non-target molecules. These fouling effects can be particularly significant during the analysis of complex clinical samples such as blood plasma, serum, or urine. There are many non-specific interactions between the surface and the complex matrix components such as hydrogen bonds, hydrophobic, electrostatic, and/or polar interactions making the reduction of the background signal a difficult task. Circulating NAs are present in very low concentrations compared to other biomolecules (e.g., proteins, lipids, etc.), further hindering their specific detection in real samples. Therefore, the development of biosensor strategies for the analysis of complex solutions directly without the need for any purification steps is on demand. Several reviews have been published describing different low-fouling coatings for label-free optical biosensors (Liu et al., 2016; Vaisocherová-Lísalová et al., 2016).

A common approach to reduce the fouling effects is the addition of different surfactants (e.g., Tween), protein-based additives (including BSA or casein), or non-protein reagents. These approaches minimize any hydrophobic and/or electrostatic attractions between the complex matrix and the functionalised surfaces. Unfortunately, they have limited antifouling properties (Vaisocherová et al., 2009). Functionalization methods should enable the immobilization of biorecognition elements and include components that provide a low-fouling background in complex solution. Hydrophilic surfaces are particularly amenable for NA hybridization because they facilitate exposure of hybridizing bases (Chen et al., 2010b). Low-fouling surfaces based on poly(ethylene glycol) (PEG) and its derivatives have been used widely to solve the problems arisen in the analyses of complex solutions (Blättler et al., 2006; Soler et al., 2014). PEG molecules create a brushed coating on the surface, which has proven to successfully prevent and reduce non-specific adsorption of proteins due to its hydrophilic properties (Soler et al., 2014). In addition, recent advances in polymer chemistry have led to the development of various polymer coatings (e.g., zwitterionic and non-ionic polymer brushes) with high resistance to fouling from complex biological fluids (Rodriguez-Emmenegger et al., 2011).

### Amplification Strategies

**Figure 3:**
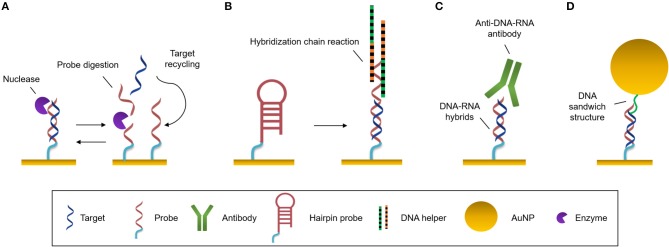
Amplification strategies. **(A)** Enzymatic reaction. Nuclease digests the probe and the target can be recycled to hybridize with another probe. **(B)** Self-catalytic reaction. Hybridization chain reaction starts when target hybridizes with the hairpin probe. It triggers the coupling of two partially complementary DNA sequences called DNA helpers. **(C)** Protein binding nucleic acids. Anti-DNA-RNA antibody recognizes DNA-RNA hybrids. **(D)** Nanomaterials. Functionalized AuNPs generate DNA sandwich structures with the hybrids.

Amplification strategies facilitate the identification and quantification of extremely low-concentrated samples or small targets which do not have enough mass, size, and/or concentration to generate a significant change in the refractive index when they are analyzed with a label-free optical biosensor. These approaches are very convenient when the sensitivity of the transducer does not range the required physiological levels. Several amplification strategies have been reviewed for optical biosensing elsewhere (Zhou et al., 2018a,b). Here we summarize some of the most employed strategies (Figure 3).

#### Enzyme-Based Amplification

This amplification approach makes use of an enzyme (most often DNA polymerase) to create a large number of copies of a specific NA. The most widely employed technique in biology is the well-known polymerase chain reaction (PCR). To amplify the analyte, it is necessary to increase the number of copies of the target sequence, increasing its concentration, prior to the interaction with the biosensor. PCR can be very effective, however it presents a number of challenges, like the requirement for sophisticated technology, for example thermal cyclers, which delay the detection times (Martzy et al., 2019). Isothermal PCR is a PCR-based strategy that is carried out at a constant temperature, increasing the speed of analysis, avoiding alternating temperature cycles. It enables higher amplification efficiencies in less amount of time compared to traditional PCR (Yoon et al., 2015). Asymmetric PCR (aPCR) preferentially amplifies one strand of the original DNA over the other, allowing selective generation of single stranded DNA (ss-DNA) amplicons by introducing one of the PCR primers at a much larger concentration during amplification (Graybill et al., 2018). These products will easily hybridize with conventional duplex DNA probes by nucleotide complementarity without requiring any DNA denature step. These different approaches are usually performed prior to hybridization of the target to the probe at the sensor surface. However, other amplification methods act once the probe is attached to the sensor surface and use it as the initiator of the amplification process. The rolling circle amplification (RCA) strategy employs padlock probes (PLPs), which are single-stranded NA probes that consist of three main parts: the 5′ and 3′ ends that hybridize with the target sequence, a sequence that interacts with the capture probes, and a universal primer binding site that generate repeated sequences. Once the target sequence is recognized, it is enzymatically joined by DNA ligase forming a circular probe-target complex. This is then amplified isothermally, generating concatenates of ss-DNA with multiple repeats of the complementary sequence (Dean et al., 2001). Xiang et al. (2015) identify *Mycobacterium* genomic DNA with a detection limit of 10 pM by employing a SPR biosensor and AuNPs for amplification.

Nucleases can be seized for signal amplification allowing recycling of the target due to the digestion of the probe. These enzymes can hydrolyse the phosphate diester bond between nucleotides, degrading NAs (Figure 3A). Ki et al. (2019) and Wei et al. (2018) used nuclease to free a DNA initiator that generate DNA sandwiches, leading a LoD for miRNA detection of 2.45 and 0.15 picoMolar (pM), respectively. In a similar way, RNase H can be used to degrade miRNA capture probes to recycle the cDNA target, amplifying the sensor signal. Ho et al. (2017) developed a biosensor to identify miR-29a which is a biomarker of influenza infection.

#### Self-Catalytic Amplification

There exist methods based on enzyme-free amplification which can achieve augment the copy number without enzymes or amplification labels, using protocols that are simpler, more stable and lower-cost. Hybridization chain reaction (HCR) is a self-catalytic amplification strategy which initiates when the target hybridizes with a complementary hairpin capture probe (Figure 3B). When the stem-loop structure of the capture probe is unfolded, it creates a one-dimensional DNA structure, and two partially complementary DNA hairpins couple with it and trigger the hybridization reaction, producing copolymers which amplify the sensor signal (Dirks and Pierce, 2004). This process can be enhanced by the use of a hairpin-free system in which ds-DNA monomers assembled into a dendritic nanostructure when it interacts with a trigger sequence which initiates the non-linear HCR (Ding et al., 2017).

Another approach consisting of Catalyzed Hairpin Assembly (CHA) has achieved an SPR signal amplification during miRNA analysis (Li et al., 2016a). Recognition of miRNA target off-chip by a hairpin (H1) triggers the interaction of the latter with a second hairpin (H2). This interaction releases the miRNA target for cyclic reuse and CHA products are generated creating more than 100-fold amplification. The main drawback from traditional CHA is the background signal generated by the non-specific CHA products when the target is not present. To overcome this limitation, mismatched base pairs can be introduced into the breathing sites of the hairpins (Li et al., 2016b).

#### Protein Binding Nucleic Acids

One of most commonly employed amplification strategies is the use of a specific antibody directed to DNA-RNA hybrids (i.e., anti-DNA-RNA antibody) (Boguslawski et al., 1986). The antibody will bind to the DNA-RNA duplexes leading to a signal proportional to the concentration of the hybridized RNA (Figure 3C). This allows for the quantification of RNA concentrations out of the range of the detection limit of the biosensor. The main advantage of the antibody is that it recognizes hybrids regardless of the sequence, allowing multiplexed measurements (Schmieder et al., 2016; Sguassero et al., 2019). This approach is of interest for the detection of short RNA sequences, which can be difficult to amplify through PCR or related methods. Also, it shows very rapid time-to-result and may enable three orders of magnitude improvement of the detection limits.

#### Nanomaterials

The high molecular weight of nanomaterials can be exploited to increase the refractive index change for each target binding event by an increment of the mass on the sensor surface. Gold nanoparticles (AuNPs) are used frequently for signal amplification in label-free optical biosensors, especially in plasmonic ones. These nanoparticles are usually functionalised with specific molecules that are able to interact with the hybridized target at the sensor surface, such as complementary DNA probes that create super sandwich structures (Figure 3D) (Vaisocherová et al., 2015; Liu et al., 2017; Melaine et al., 2017) or anti DNA-RNA hybrid antibody (Sguassero et al., 2019). Gold nanoparticles not only increase the mass, but it has also been shown that the local SPR of the particle can lead to further increase in the apparent effective refractive index due to a phenomenon called coupling effect. The coupling effect is a distance dependent factor, achieving maximum enhancement within 8 nm distance of the particle from the surface for 20 nm diameter AuNPs. Outside this distance, the size/mass properties are the major factors in the signal improvement (Hong and Hall, 2012). Similar properties have been demonstrated with gold nanorods, enabling SPR signal enhancement for the detection of miRNAs at the femtomolar level (Hao et al., 2017). In addition, graphene sheets can be enriched with AuNPs, forming graphene oxide-AuNPs composites (GO-AuNPs) that can offer the physicochemical properties of both materials. Additionally, GO-AuNPs can be used in two ways: as sensor substrate, providing a high surface area to significantly increase the immobilized bioreceptors; and as an amplification element (Li et al., 2017). Gold nanoparticle-decorated molybdenum sulfide (AuNPs-MoS_2_) can also be created by enriching the edge or defective sites of MoS_2_ sheets with gold nanoparticles (Nie et al., 2017).

## Microfluidics Systems for NA Analysis

The unprecedent development of label-free optical biosensors due to technological evolution from macro, to micro,- and nanotechnologies has been further enhanced by integration with microfluidic circuits. Conventional microfluidic devices rely on the continuous flow of liquids in channels of a few microns usually fabricated using soft-lithography methods (Luka et al., 2015). They are mostly fabricated in polymer material such as polyethylmethacrylate (PMMA) and polydimethylsiloxane (PDMS) due to their excellent chemical, physical, and mechanical properties, and excellent biocompatibility. Microfluidics have been harness for label-free NA detection, and is a very promising platform for parallelization of analyses, increasing the flow-throughput of the sample while minimizing sample volumes, which significantly reduces the cost of the assays (Baratchi et al., 2014). Microfluidics has enabled the label-free detection of different NAs such as mRNAs (Huertas et al., 2017) or miRNAs (Graybill et al., 2018) in multiplexed devices by the creation of independent parallel channels that allow sample delivery over specific biosensors.

In addition, different microfluidic designs can be fabricated on a single chip and disposed in customized arrangements to perform different procedures (Jung et al., 2015). This is of particular interest in NA analyses, since protocols usually involve multiple steps, such as sample lysis to expose NAs found in the cells or exosomes, their purification and extraction or their amplification to increase the target copy number before the eventual detection by the biosensors, as discussed in the previous section. mRNA isolation has been achieved by the purification with oligo (dT) that selectively hybridize to the poly (A) tail found mRNAs, achieving up to 70% efficiency (Satterfield et al., 2007). The functionalised photopolymerized monolith was employed to purify mRNA from eukaryotic cell total RNA. An RNA isolation microfluidics have been developed capable of obtaining high-quality RNA from different biological samples within 30 min (Yoon et al., 2018). This device took advantage of the capability of dimethyl adipimidate agent to bind to RNA in a pH-dependent and reversible manner, avoiding the employment of chaotropic salts or spare solvent. The non-chaotropic nature of this molecule allowed to simplify the isolation process and highly reduced any contamination derived from the conventional methods, enhancing the quality of the extracted RNA (≈87% recovery rate). Similarly, non-chaotropic agents can be applied for DNA extraction and the potential of this methodology has been demonstrated for the analysis of genetic and epigenetic alterations (Shin et al., 2014), leading to high quality and quantity of DNA purification from urine and blood samples. The high quality of the extracted material enables further on-chip amplification through PCR-based methods to increase the sensor sensitivity. Haber et al. (2017) developed a microfluidic platform for qPCR integrated with an LSPR biosensor. The microfluidic PCR chip utilized piezoelectrically-pumped recirculating flow. They demonstrated multiplexed detection of *E. coli* DNA target amplification within 15 min, achieving a LoD of 5 fg/μL. Also, an integrated microfluidic PCR device and an SPR-fiber sensor has allowed DNA amplification of *Salmonella* spp. and its downstream label-free detection (Nguyen et al., 2017). In this case, a serpentine micro-channel was fabricated over two copper heat blocks, allowing on-chip thermal cycling.

These findings highlight the potential of combining label-free optical biosensors with different microfluidic modules for a faster and more user-friendly operation. In addition, automated microfluidics for NA analyses can be achieved by incorporating a series of microvalves and micropumps (Kim et al., 2012). These automated circuits have been proven to be of considerable value when integrated to multiplexed photonic chips (Szydzik et al., 2017), presenting considerable advantages in the fabrication of LoC platforms (Jung et al., 2015). All in all, microfluidic integration empowers label-free optical biosensors with exceptional capabilities and contribute to the development of portable and user-friendly devices which can be used at the doctor's office or the patient's home (González-Guerrero et al., 2016; Lopez et al., 2017).

## Applications for Clinical Diagnosis

### DNA Punctual Mutations

SNPs are produced by the change of a single nucleotide for another one within a gene sequence. This means that the translation process from mRNA to protein might suffer some alteration resulting in no translation of a protein, incorrect folding or translation of a non-functional protein. The percentage of SPNs in human samples is typically very low and the specificity required should have resolutions at the single nucleotide level. Therefore, in the last years, the efforts have been focused in increasing the sensitivity and specificity of SNP analysis through different label-free optical biosensor approaches in a faster and less laborious manner (Table 2). Nguyen and Sim (2015) used an LSPR based on scattered Rayleigh light from gold nanoparticles to identify E545K and E542K mutations in *PIK3CA* gene from ctDNA. They biofunctionalized gold nanoparticles with a specific PNA to promote the specificity at the single mismatch level, which is an important requisite for NA detection. These PNA hybridized 100% with ctDNA and mismatched with normal circulant DNA, achieving a limit of detection of 200 fM. In addition, this methodology also enabled recognition of the DNA methylation profile of the captured sequences by immunogold colloid functionalised with anti-5-methylcytosine (anti-5-mC) antibody, obtaining an enhanced LSPR resonant signal only from the methylated DNA due to the presence of the immunogold colloids. Through this secondary detection approach, they were able to identify 2 mCpG sites on the ctDNA and improve the detection limit by a factor of four. Another LSPR biosensor was developed to compare the hybridization rates of perfectly matched and mismatched sequences that only differed in one nucleotide in codon 12 of *KRAS* gene (Rapisarda et al., 2017). In this study, SH-DNA probes were employed for a direct and label-free detection of the sequences, achieving LoD of 10 and 13 nM, respectively. They also evaluated the hybridization rates from a mix of perfect/mismatched sequences with different ratios. The non-specific interactions of the mismatched sequence with the capture probe suggested that the development of different bioreceptors, such as PNA or new bioreceptors could improve the specificity and the selectivity. Indeed, a new neutralized chimeric DNA analog bioreceptor was developed to detect perfect matched sequences, discriminating mismatched ones (Huang et al., 2018). The chimeric DNA probes contained four methylated nucleotides in the backbone for charge neutralization and a NH_2_ group in the 5'-end for its immobilization in a glutaraldehyde-activated SAM. By using an SPRi biosensor, they evaluated the efficiency of this new bioreceptor to discriminate sequences that differed in a single nucleotide, demonstrating a favorable hybridization of perfectly matched DNA sequences with their chimeric probe under specific conditions of temperature and ionic strength. The activity of this chimeric DNA could be potentially enhanced by carefully designing probes sequences with different methyl sites.

**Table 2:** Optical biosensors for DNA punctual mutations detection: mutations, genes involved, real samples analyzed, and limit of detection achieved.

**Sensor**	**SNP**	**Sample**	**LoD**	**Amplification**	**References**
LSPR	E545K and E542K (*PIK3CA)*	ctDNA in plasma	200 fM 50 fM	Immunogold colloids	Nguyen and Sim, 2015
LSPR	codon 12 of (*KRAS)*	Buffer	10 nM and 13 nM	NA	Rapisarda et al., 2017
Micro-ring resonators	G12D and G13D in KRAS	Colorectal cancer tissue	1% mutant allele	ISAD	Jin et al., 2017
MZI	L858R in *EGFR*	Lung cancer tissue	1% mutant alleles	ISAD	Liu et al., 2015b

**Figure 4:**
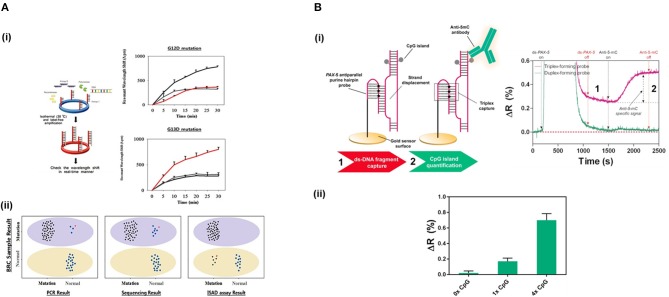
Detection of SNPs and methylation profile with optical biosensors. **(A)** Detection of two SNPs in *KRAS* gene using silicon microring resonators sensors. (i) ISAD-KRAS assay representation and calibration curves reflecting the resonant wavelength shift obtained for G12D and G13D mutants of *KRAS* gene (gray: wild-type, black: G12D mutant, red: G13D mutant). (ii) Validation of the ISAD-KRAS assay in 70 clinical samples of colorectal cancer patients (50 mutants and 20 wild-type) for G12D and G13D mutations, and comparison with conventional techniques as PCR and sequencing. Purple oval represents the mutant area and yellow oval the wild-type one. The mutant allele (black) and wild allele (blue) are also shown. Readapted with permission from Jin et al. (2017). Copyright © 2017 Jin et al. Creative Commons Attribution License 3.0 (CC BY 3.0). **(B)** Detection of the methylation profile of *PAX-5* gene using a SPR biosensor. (i) Scheme and real-time recognition of the two-step assay developed for the detection of (1) ds-DNA fragments and (2) 5'methyl cytosines by the PPRH probe and specific anti-5-mC antibody, respectively. (ii) Identification of different methylation profiles (0x, 1x, and 4x 5' methyl cytosines). Reprinted from Huertas et al. (2018), Copyright (2018), with permission from Elsevier.

Jin et al. (2017) developed a micro-ring resonator sensor combined with isothermal solid-phase amplification/detection (ISAD) to identify G12D and G13D mutations found in codon 12 and codon 13 of *KRAS* gene from 70 colon rectal cancer tissue samples (Figure 4A). The sensor surface was functionalized with G12D or G13D mutant primers followed by an on-chip ISAD. Since only the mutant primers were immobilized, mutant alleles were specifically amplified from the clinical samples while wild-type ones displayed no sensor signal. The ISAD-KRAS approach showed more specificity and selectivity than PCR and direct sequencing methods, detecting samples containing only 1% of the mutant allele compared to the 30% shown by these techniques. By evaluating 70 cases of colorectal cancer, ISAD-KRAS detected even cases where PCR and direct sequencing did not have sufficient sensitivity. A similar approach was followed by Liu et al. (2015b) to identify punctual mutations in the *EGFR* gene, specifically, the L858R mutation. They identified 1% of mutant alleles in a sample of mixed cellular types in contrast with the 25% achieved by PCR. Moreover, they evaluated lung cancer patient samples, correctly detecting the L858R mutation in samples that were also detected by direct sequencing. It was also possible to identify cases that the PCR could not detect due to the low amount of DNA. Both ISAD-based approaches have been demonstrated to be promising diagnosis tools for SNPs analyses and appealing alternatives to PCR and sequencing methods due to their fast operation time (<30 min), sensitivity, specificity, and cost-effective relation.

### DNA Methylation Profile

DNA methylation is an epigenetic mark found in some cytosines generally located in CpG islands of most promoter regions. The level of methylation of these regions determines the accessibility of the transcription factor to the promoter and, therefore, the levels of expression of the genes. Normally, the hypermethylation of these CpGs regions are related to down-transcription and, inversely, hypomethylation means overexpression (Rodriguez et al., 2006). The detection of the methylation status using label-free optical biosensors has attracted increasing attention over the last few years (Nazmul Islam et al., 2017). As per SNPs, the concentration of these biomarkers is rather low in samples (Gai and Sun, 2019) (Table 3). In addition, they should be able to specifically detect the methylation status at the single cytosine resolution for more accurate analyses. Yoon et al. (2015) developed a LoC device based on micro-ring resonators and bisulfite conversion methodology. Bisulfite is a carcinogenic compound which converts the non-methylated cytosines into uracil whereas methylated cytosines remain intact. A pre-processing module including a microchamber for bisulfite treatment, a micromixer that passively mixed the sample, and a DNA-purification microchannel were integrated on the same chip and coupled to a detection module. After the purification step, an ISAD was performed using methyl- and unmethyl-specific primers grafted in micro-ring resonators for the analysis of the methylation profile of *RAR*β gene from genomic DNA of MCF-7 cells. Methylated sequences produced a shift in the resonant wavelength of the resonator immobilized with methyl-specific primers, whereas no amplification was observed in the resonator functionalised with the unmethyl-specific ones. The described LoC enabled identification of methylation levels of 1% in a mix of methylated/unmethylated DNA compared to the 10% achieved by RT-PCR. It also eliminated the human errors introduced during the manual bisulfite treatment and could identify the DNA methylation status in only 80 min of analysis, in contrast with conventional methods (24 h). However, it did not suppress the long and intense processes related to the bisulfite conversion. In addition, bisulfite conversion cannot be used to identify other epigenetic marks generated by enzymatic oxidization and that are highly important for the assessment of the methylation process, such as of 5-mC, such as 5-hydroxymethylcytosines (5-hmC), 5-formylcytosine (5-faC), and 5-carboxylcytosine (5-caC) (Chowdhury et al., 2014). Due to this lack of versatility, other detection approaches have been pursued.

**Table 3:** Optical biosensors for DNA methylation profiles identification: genes involved, real samples analyzed, methodology employed, and detection limit reached.

**Sensor**	**Gene**	**Sample**	**Methodology**	**LOD**	**Amplification**	**References**
SPR	Synthesized ODN	genomic λ DNA and HCT116 human colon cancer cells	biotinylated bulge inducer probe/ anti-5-mC	1 cytosine in 48 fg	NA	Kurita et al., 2015
SPR	Synthesized *PAX-5* gene	Buffer	poly-purine reverse-Hoogsten hairpin/anti-5-mC	Single-cytosine	NA	Huertas et al., 2018
SPR	*MGMT* promoter gene	genomic λ DNA	alkylating linker molecule/anti-5-mC	25%	NA	Kurinomaru et al., 2017
LSPR	*p53* gene promoter	DNA extraction of HeLa and HEK 293 cells	PNA/methyl-CpG binding domain protein 2	Single-cytosine	NA	Nguyen et al., 2015
Micro-ring resonators	*RARβ* gene	genomic DNA of MCF-7 cells	Methylated/unmethylated primers	1% in methylated/ unmethylated mixture	ISAD	Yoon et al., 2015
Toroidal resonant cavity	Synthesized ODN	Buffer	Anti-5-hmC	0.42 pM	NA	Hawk and Armani, 2015

Methyl-cytosines and their derivatives can be specifically analyzed by the employment of specific antibodies and proteins. Kurita et al. (2015) designed a microfluidic device to measure DNA methylation in an SPR sensor. They mixed fragmented DNA with a biotinylated bulge inducer probe, exposing the cytosine in a looped-out conformation. Then, this DNA-bulge complex was attached on a streptavidin surface by streptavidin-biotin interaction. Finally, they identified the methylated cytosine using an anti-5-mC antibody, achieving single-cytosine resolution in 48 fg of genomic HCT116 human colon cancer cells. The simplicity and miniaturization of the microfluidic sensor offer great advantages for this methodology. Nevertheless, a denaturalization step off-chip is necessary to allow the target hybridization with the bulge inducer probe, which leads to an increase in the detection time. Different approaches have been developed in order to directly capture intact ds-DNA targets without the need for denaturalization steps, resolving the problem of the direct analysis of ds-DNA molecules. DNA methylation status has been analyzed by the direct immobilization of ds-DNA on gold surfaces by employing an alkylating linker molecule, L1 (Kurinomaru et al., 2017). This molecule consists of two reactive groups, nitrogen mustards, that interacts with DNA, and cyclic disulphides, attaching to the gold substrate. Using an SPR biosensor, they directly immobilized methylated ds-DNA on the gold sensor surface and assessed the methylation levels employing anti-5-mC antibody. This approach could be a promising tool to decipher global methylation status but lacks specificity for concrete DNA methylation sequences. Much of hybridization-based analytical techniques struggle to capture ds-DNA due to the use of probes which analytical performances are limited to duplex formation. Alternative approaches have been implemented to overcome such limitation. Nguyen et al. (2015) employed an LSPR biosensor based on gold nanostars and Rayleigh resonance to assess the methylation profile of *p53* gene promoter. They biofunctionalized the gold surface with a specific PNA, which enables to form stable complexes with ds-DNA. They quantified the methylation status of the captured ds-DNA target by specific interaction with methyl-CpG binding domain protein 2. This biosensor achieved a limit of detection down to the single-cytosine and it was applied to DNA samples from HeLa and HEK 293 cells-extraction. Moreover, it allowed the study of conformational changes of *p53* gene promoter and the relative transcriptional efficiencies regulated by methylation, highlighting its potential as a tool to study the effects generated by the presence of this epigenetic mark. Huertas et al. (2018) also achieved a direct detection of ds-DNA and the evaluation of methylation status employing an SPR biosensor within 30 min. They used a novel bioreceptor based on poly-purine reverse-Hoogsten hairpin (PPRH) probes that permitted the direct recognition of ds-DNA by inducing strand displacement and promoting triple-helix formation (Figure 4B). The PPRH probe presented 8-aminoG modifications to enhance the strength of the capture, which further increased the stabilization of the triple helix structure. The efficient capture of ds-DNA fragments allowed to quantify and assess different methylation profiles (from 1 to 4 cytosines) in *PAX-5* gene promoter through immunodetection using anti-5-mC antibody.

DNA methylation analysis based on immunodetection allows for the investigation of other epigenetic modifications rather than 5-mC, which can be detected by employing the specific antibodies for each one. Hawk and Armani (2015) employed a toroidal resonant cavity sensor for the identification of DNA containing 5-hydroxymethylcytosines by using a specific anti-5-hmC antibody. In this case, instead of using a DNA probe, they attached the antibody to the sensor surface. Hence, direct detection of synthetized ds-DNA with methylated, unmethylated and hydroxymethylated epigenetics modifications was carried out, showing a limit of detection of 0.42 pM and demonstrating the versatility of the assay for different epigenetic modifications.

### Long RNAs

Long RNAs are characterized by a length from 200 bases up to several kilobases. They can be classified as coding RNAs and non-coding RNAs depending on if they encode the information (i.e., mRNA) for the synthesis of proteins or not. The latter ones have structural [transfer RNA (tRNA), ribosomal RNA (rRNA), small nuclear RNA (snRNA)] and regulatory roles [Long intergenic non-coding RNAs (lncRNAs); Natural Antisense Transcripts; Promoter associated ncRNAs, etc.] (Carrascosa et al., 2016). One advantage of long-RNA biosensing is the large refractive index changes they produce due to their large mases, which is translated to higher sensor signal intensities. Table 4 summarizes different strategies for long RNA biosensing.

**Table 4:** Optical biosensors for long RNAs detection: type of RNA, real samples analyzed, strategies to improve the sensitivity, and the demonstrated limit of detection.

**Sensor**	**RNA**	**Sample**	**LOD**	**Amplification**	**References**
SPR	*Fas57/Fas567* mRNA alternative splicing	HeLa cell total RNA	387/438 pM	NA	Huertas et al., 2016a
SPRi	16S rRNAs	*L. pneumophila, P.auriginosa* and *S.typhimurium* total RNA	10 pg/mL	AuNPs	Melaine et al., 2017
Micro-ring resonator	lncRNAs KIAA0495/PDAM and MALAT1	Patient-derived xenograft GBM6 RNA	C(t) values	aPCR	Cardenosa-Rubio et al., 2018
BiMW	*Fas57/Fas567* mRNA alternative splicing	Buffer	580/735 fM	NA	Huertas et al., 2017

#### Messenger RNAs

**Figure 5:**
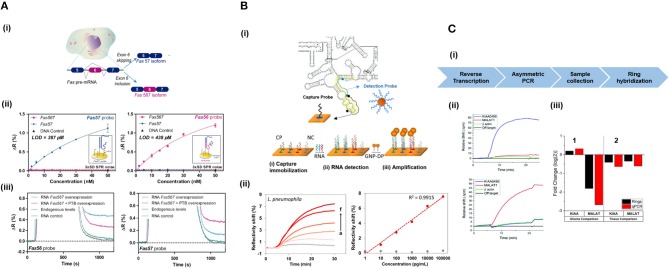
Detection of long RNAs with optical biosensors. **(A)** Quantification of alternatively spliced mRNA isoforms from *Fas* gene using a SPR biosensor. (i) Scheme of the different RNA isoforms generated by alternative splicing of *Fas* gene. (ii) Calibration curves for *Fas57* and *Fas567* isoforms and scheme of the DNA-probes used for the hybridization. (iii) SPR sensograms of the detection of total extracted HeLa cell RNA for Fas56 (left) and Fas57 (right) probes. Reprinted from Huertas et al. (2016a), Copyright (2016), with permission from Elsevier. **(B)** Detection of 16S rRNA from *Pseudomonas aeruginosa, Salmonella typhimurium*, and *Legionella pneumophila* using a SPRi sensor. (i) Capture probes (CP) are immobilized for the hybridization of RNA and amplification is performed by using nanoparticles functionalized with the detection probes (GNP-DP). Probe strategy design are illustrated using 16S rRNA sequence of *Pseudomonas aeruginosa* as an example. (ii) SPR sensograms and calibration curves for quantification of *L. pneumophila* RNA strain using amplification. Adapted with permission from Melaine et al. (2017). Copyright © 2017 American Chemical. **(C)** Detection of KIAA0495 and MALAT1 lncRNAs with microring resonator arrays. (i) Scheme of the overall assay for lncRNA detection. (ii) Identification of each lncRNA and evaluation of the specificity using β-actin as internal control. (iii) Assessment of lncRNAs expression in GBM6 cells compared to conventional, single-plex RT-qPCR technique. Healthy brain (1) and lung (2) tissues were used as reference. Republished with permission of The Royal Society of Chemistry from Cardenosa-Rubio et al. (2018); permission conveyed through Copyright Clearance Center Inc.

mRNA analysis gives information about the translation of a particular protein, allowing even the identification of the cell types that are suffering changes in the translation process that might be related to the beginning of some forms of diseases. Discrimination among the diverse mRNAs transcripts is essential. Alternative splicing process can generate different mRNA isoforms from a common pre-mRNA. These isoforms share common exon sequences that might result in cross-hybridization. For all these reasons, a small number of label-free optical biosensors have been developed for monitoring alternative splicing processes for diagnostic purposes. Huertas et al. (2016a) developed an SPR biosensor for the direct quantification of *Fas* gene alternative splicing mRNA isoforms (*Fas57/Fas567*, Figure 5A). Prior to detection, they optimized a fragmentation step based on RNA alkaline hydrolysis to customize the isoforms' length to the biosensor convenience (≈200-bases fragments). This strategy allowed to overcome the challenge encountered in the detection of long RNA molecules and open the door to explore new biomarkers in evanescent-wave optical biosensors based on long RNA targets. For detection, they immobilized SH-DNA probes on the gold sensor surface containing the splice-junction sequences, the key feature for the specific recognition of each isoform. Also, they optimized a highly stringent buffer by adjusting with the formamide content and the ionic strength, achieving 100% selectivity and a limit of detection of 387/438 pM for each isoform. The methodology was validated by evaluating purified RNA samples from different HeLa cell lineages, which were in good agreement with conventional RT-qPCR-based methodology. In addition, further implementation in a multiplexed BiMW biosensor outperformed the SPR biosensor, exhibiting LODs with three order of magnitude improvement thanks to the high sensitivity of these interferometric biosensors (Huertas et al., 2017).

#### Ribosomal RNAs

Ribosomes are hybrid complexes formed by proteins and rRNA sequences that forms part of a large ribosomal subunit and a small one. Although the different subunits have highly conserved molecular weights and sequences, they differ among organisms, enabling creation of phylogenies and identification of the microorganisms. rRNA is present in high concentrations inside the cell, but its secondary structure may hinder the recognition event by DNA probes, limiting the sensitivity. Melaine et al. (2017) used a SPRi sensor for the simultaneous detection of 16S rRNAs (a component of the small ribosomal subunit in procariotes) from three pathogenic bacterial strains: *L. pneumophila, P. aeruginosa*, and *S. typhimurium* (Figure 5B). They selectively captured the targets by duplex DNA probes at the sensor surface. Subsequently, they performed a signal amplification based on Au-NP functionalized with a DNA probe specific to a second target region of the previously captured rRNA sequences. The amplification approach resulted in a 3-fold increase of the SPR signal, achieving a limit of detection of 10 pg/mL and a range of detection of 0.01–100 ng/mL, which covers the wide dynamic range of recognition necessary for the detection of the NA expression dynamics as highlighted in the challenges. Total RNA from the different bacterial cultures were analyzed after RNA extraction and fragmentation by a multiplex approach that allowed to detect in a selective and fast way 16S rRNA from the three different bacterial strains.

#### Long Non-coding RNAs

The first lncRNA discovered and studied was H19 (Huang et al., 2019). Since then, many additional lncRNAs have been identified and investigated as they interfere in several biological functions. For example, KIAA0495/PDAM can act as tumor suppressors in oligodendrioglioma while MALAT1 (metastasis associated lung adenocarcinoma transcript 1) is related to different tumors being able to act as tumor suppressor in glioblastoma. Cardenosa-Rubio et al. (2018) used a micro-ring resonator for the multiplexed detection of the three lncRNAs (Figure 5C). They carried out a PCR amplification prior detection, allowing the subsequent hybridization of the generated ss-DNAs with complementary DNA probes spotted in array distribution in the silicon sensor surface. The selected lncRNAs were tested in spiked-in commercial brain and lung total RNA samples and in RNA from glioblastoma cell line (GBM6). Results were validated by RT-qPCR and showed high consistency with results from precedent literature.

### Short Non-coding RNAs

Small RNAs are characterized by a short length around 20–200 bases and they englobe microRNAs (21–25 bases), piwiRNAs (20–30 bases), tinyRNAs (<22 bases) and small interfering RNAs (20–25 bases). All play a structural or regulatory role so they are called small non-coding RNAs because they are not translated into proteins (Carrascosa et al., 2016). Most label-free optical biosensors have been harnessed for the detection of miRNAs (Table 5). Schmieder et al. (2016) employed an SPR biosensor to detect miRNA-93. To maximize both, the selectivity and sensitivity of the SPR detection, they used a SH-LNA probe and subsequent amplification by anti-DNA/RNA hybrid antibody, enhancing the sensor response by one order of magnitude. Enhanced amplification can be achieved by the combination of this antibody with Au-NPs. Sguassero et al. (2019) introduced a multiplexed SPRi biosensor for the detection of four miRNAs potentially involved in multiple sclerosis. The antibody permitted the recognition of the different miRNA sequences selectively hybridized on the sensor surface, producing an enhanced SPRi signal correspondent to the concentration of captured miRNAs. This approach was used to analyse to RNA extracted from blood samples from multiple sclerosis patients and succeeded in detection of relevant miRNAs at the sub-picomolar range.

**Table 5:** Optical biosensors for miRNA detection: strategies to improve the sensitivity, the demonstrated limit of detection and the real samples analyzed.

**Sensor**	**Target miRNA**	**Sample**	**LOD**	**Amplification**	**References**
SPR	miR-145	Buffer	1 nM	NA	Aviñó et al., 2016
SPR	miR-93	Buffer	10 pM	Antibody	Schmieder et al., 2016
SPR	miR-141	total RNA extracted from cancer cell lines (prostate: 22Rv1; hepatocellular:SMMC7721; colon LoVo)	0.1 fM	GO-AuNPs	Li et al., 2017
SPR	miR-21	Hepatocarcinoma cell line (Bel-7404); SMMC-7721; L-02; Hela	0.6 fM	AuNPs	Liu et al., 2017
SPR	miR-141	total RNA extracted from cancer cell lines (prostate: 22Rv1; hepatocellular: SMMC7721; colon LoVo); 10 % human serum	0.5 fM	AuNPs-MoS2	Nie et al., 2017
SPR	miR-21	total RNA extracted from MCF-7 cells	1 pM	CHA	Li et al., 2016b
SPR	miR-21	total RNA extracted from MCF-7 cells	< 1 pM	CHA	Li et al., 2016a
SPRi	miR-21 and miR-192	Buffer	0.15 pM (miR-21) and 0.22 pM (miR192)	Nuclease + AuNPs	Wei et al., 2018
SPRi	miR-422; miR-223; miR-126; miR-23a	total RNA extracted from blood	0.5 pM	AuNP + antibody	Sguassero et al., 2019
SPRi	miR-29-3p	total RNA extracted from throat swab		RNase H	Ho et al., 2017
LSPR	miR-10b	urine and plasma samples of mice with orthotopic Hs746tT xenografts	2.45 pM	Nuclease + HCR + tannic tags	Ki et al., 2019
Mirroring resonator	miRNA-let7f, miRNA-219, miRNA-10b, miRNA-29a, miRNA-335, miRNA-124a, miRNA-222, miRNA-34a, miRNA-155	RNA extracted surgical glioma cells	Ct	aPCR	Graybill et al., 2018
MZI	miR-21, let-7a	urine (bladder cancer) and cell lines (MCF7 and A549)	1 nM	NA	Liu et al., 2015a
BiMW	miR-181a	Urine (bladder cancer)	23 aM	NA	Huertas et al., 2016b

**Figure 6:**
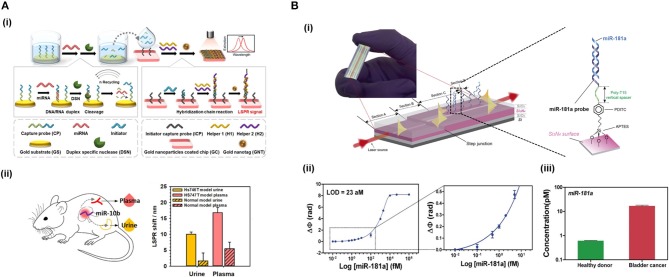
Detection of miRNAs by optical biosensors. **(A)** Detection of miR-10b for LSPR-based biosensor. (i) Schematic representation of miRNA detection using duplex-specific nuclease for recycling and hybridization chain reaction with tannic acid tags for amplification. (ii) LSPR shifts in urine and plasma mice with orthotopic Hs746tT xenografts samples compared to normal model mice. Reprinted with permission from Ki et al. (2019). Copyright © 2019, American Chemical Society. **(B)** Detection of miR-181a using a BiMW interferometer. (i) Working principle of the BiMW interferometer and biofunctionalization strategy. (ii) Calibration curve of miR-181a in semilog scale. (iii) miR-181a quantification in urine samples from healthy donors and bladder cancer patients. Adapted with permission from Huertas et al. (2016b). Copyright © 2016, American Chemical Society.

The versatility of label-free optical biosensors allows integration of different amplification methods within the same assay. Wei et al. (2018) employed an SPRi biosensor for the multiplexed assessment of miR-21 and miR-192 in buffer. In order to increase the sensitivity, they integrated strand displacement amplification (SDA) with gold nanoparticles. To do so, they designed hairpin probes for the amplification that consisted of three different domains, including the miRNA-recognition sequence, an amplification domain and a common recognition domain which hybridizes with both, a SH-DNA probe immobilized at the sensor surface and the gold nanoparticles. The different miRNAs interacted specifically with their recognition sequence in each amplification probe, causing the opening of the hairpin probe loop and initiating the SDA. Finally, the released triggers hybridized with the captured probes on the sensor surface and the DNA functionalized AuNPs, producing a strong change in mass with a correspondingly large signal. This method was capable of simultaneously detecting miR-21 and miR-192 at the low picomolar range in 10% diluted bovine serum. An analogous strategy was developed by Ki et al. (2019) (Figure 6A). They used an LSPR biosensor based on gold nanostars to detect miR-10b in urine and plasma samples from mice with orthotopic Hs746tT xenografts. Amplification was performed by duplex-specific nuclease that degraded the DNA probe in DNA/RNA hybrids, recycling the target and liberating a DNA initiator. DNA initiator was captured creating a DNA sandwich with DNA helpers and tannic acid gold nanotags, allowing 2.42 pM LoD. Results showed that miR-10b concentration was higher in plasma than in urine samples.

Dual amplification was achieved by the generation of two layers of GO-AuNPs composite in an SPR sensor (Li et al., 2017). The bottom layer served as a surface for the immobilization of SH-DNA probes on the nanoparticles, providing functionality to the sensor. In a second step, the target miRNA was hybridized with the immobilized probes and subsequently interacted with DNA functionalized GO-AuNPs composites, forming the upper layer that acted as a enhancer of the sensor signal. They showed one order of magnitude LOD improvement in the two-layer approach compared to the performance of the single layer used as a substrate, achieving extremely sensitive detection (0.1 fM) of miRNA-141 extracted from different cancer cell lines. In addition, they obtained corresponding levels of expression with RT-qPCR. A similar strategy was developed based on gold nanoparticles-decorated molybdenum sulfide (AuNPs-MoS2) as amplification element (Nie et al., 2017). SH-DNA probes complementary to miR-141 were immobilized on an SPR biosensor to identify miR-141 in the same cellular lines and HeLa cells, achieving a limit of detection of 0.5 fM. Moreover, they assessed the accuracy in spike samples diluted in 10% human serum acquiring reliable values validated by RT-qPCR.

Other methods to increase the sensitivity during miRNA detection are based on probe design. Aviñó et al. (2016) synthetized a new DNA tail-clamp purine track to detect miR-145. This probe promoted a triple helix with the RNA target, improving 2.4 times the sensitivity over duplex forming probes. In addition, an 8-Amino-2′-deoxyguanosine modification was introduced to stabilize the triplex by 1.5 times, making this new probe attractive for improved miRNA detection.

Graybill et al. (2018) employed a micro-ring resonator array to analyse eight miRNAs from surgical glioma cells. They amplified the signal using aPCR and evaluated the hybridization rate of ss-DNA as Ct, like qPCR, achieving values of level expression similar to the literature for surgical glioma cells. Also, a MZI was employed for the detection of miRNAs by Liu et al. (2015a). They identified two miRNAs (miR-21 and let-7a) that participate in bladder cancer onset in human urine samples and obtained a LOD of 1 fmol/μL (1 nM). In addition, they identified SNPs from let-7 family miRNAs. Finally, attomolar detection limit of miR-181 (LoD = 23 aM) in a direct assay (<20 min) was achieved using a BiMW interferometer (Huertas et al., 2016b) (Figure 6B). The ultra-high sensitivity achieved reveals the potential of these types of optical transducers for the analysis of extremely low concentrations with no need for any amplification strategy. They discriminated homologous miRNAs at single nucleotide mismatched, as well as pre-miRNA-181, during the evaluation of miR-181 in urine samples from cancer bladder patients. These results uncovered the participation of this miRNA in the development of bladder cancer.

## Conclusions and Future Perspectives

The employment of evanescent-wave optical biosensors for the routine analysis of NA-based biomarkers threatens to change the concept of diagnosis. The opportunity for fast, highly sensitive, and multiplexed analyses of the genetic and epigenetic landscape of different diseases makes these devices a very attractive diagnostic solution. Numerous biosensing approaches have been proposed as solutions for use in clinical environments. Thanks to their highly sensitive transducers and their ease to be combined with different amplification methods, label-free optical biosensors have allowed NA detection in a wide dynamic range of concentrations, from nanomolar down to attomolar. Their versatility to incorporate specialized probes and immunodetection methods have also enhanced the selectivity of the analysis for the identification of a great variety of NA biomarkers, including SNPs, methylation patterns, mRNAs, and miRNAs. In addition, these sensors are well-suited to integration within lab-on-a-chip microsystems, enabling further enhancement through combination with a variety of pre- and post-processes, thanks to monolithic integration with complex and automated microfluidic systems, ultimately enabling the development of low-cost, multiplexed, and user-friendly platforms.

Unfortunately, there still exist some drawbacks to be surpassed. Most of the applications described in this work are preclinical proof-of-concepts performed in the very controlled laboratory environment environments. This can difficult the complete translation of the technology to real clinical settings. Further investigations need to be fulfilled to confirm their feasibility which should be aligned with internationally established guidelines. Efforts should be focused on the development of biosensors capable of simultaneous multiplexed analyses in a few minutes. In terms of integration, new microfluidic approaches should be implemented to meet the specific pre- and post-processing requirements for each biomarker inside the same sensor platform guaranteeing and testing their stability and reproducibility. In addition, sample volumes should be reduced to few microliters.

On the other hand, the vast genetic and epigenetic network makes calls for a new system for well-defined biomarker panels linked to concrete diseases and disease stages. Extensive testing in clinical trials is required to establish standard patterns for each case. Future trends should be focused on leveraging integrated microfluidics and multiplexed label-free optical biosensors to create highly complex diagnostic, monitoring and prognostic systems. These complex systems will unlock even more detailed and specific information. The sheer vastness and subtlety of the form and function of the NA biomarkers suggest that big data and bioinformatic analysis approaches will be required to extract useful information from these analyses. Such an endeavor will contribute to making connections between specific diseases and signatures. Interactivity in the form of potentially reversible epigenetics will add an additional dimension.

On the whole, the analysis of genetic and epigenetic biomarkers is a key requirement for the effective analysis and understanding of important malignancies, such as infectious diseases and cancer. The potential reversibility of genetic and epigenetic mechanisms poses great promise in the development of personalized treatments for each patient. Indeed, in precision medicine, the aim is the molecular characterization of each patient, which can only be realized by the employment of low-cost and high throughput screening technologies. There is a lot that can be gleaned on patient-derived data with PoC devices, which can be very valuable. Such a holistic approach can also be of value for personalized drug delivery, effectively provide tailored medications and therapies, ensuring that the most adequate treatment is given to the right patient with a proper dose at the correct time.

## Author Contributions

CH and LL devised the review. CH and OC-L conducted the literature review and provided the first draft. CH and OC-L created the figures. CH, OC-L, AM, and LL contributed to manuscript revision and approved the submitted version.

### Conflict of Interest

The authors declare that the research was conducted in the absence of any commercial or financial relationships that could be construed as a potential conflict of interest.
